# GRXCR2 Regulates Taperin Localization Critical for Stereocilia Morphology and Hearing

**DOI:** 10.1016/j.celrep.2018.09.063

**Published:** 2018-10-30

**Authors:** Chang Liu, Na Luo, Chun-Yu Tung, Benjamin J. Perrin, Bo Zhao

**Affiliations:** 1 Department of Otolaryngology-Head and Neck Surgery, Indiana University School of Medicine, Indianapolis, IN 46202, USA; 2 Department of Biology, Indiana University-Purdue University Indianapolis, Indianapolis, IN 46202, USA; 4 Lead Contact

## Abstract

Mutations in human *GRXCR2*, which encodes a protein of undetermined function, cause hearing loss by unknown mechanisms. We found that mouse GRXCR2 localizes to the base of the stereocilia, which are actin-based mechanosensing organelles in cochlear hair cells that convert sound-induced vibrations into electrical signals. The stereocilia base also contains taperin, another protein of unknown function required for human hearing. We show that taperin and GRXCR2 form a complex and that taperin is diffused throughout the stereocilia length in *Grxcr2*-deficient hair cells. Stereocilia lacking GRXCR2 are longer than normal and disorganized due to the mislocalization of taperin, which could modulate the actin cytoskeleton in stereocilia. Remarkably, reducing taperin expression levels could rescue the morphological defects of stereocilia and restore the hearing of *Grxcr2*-deficient mice. Thus, our findings suggest that GRXCR2 is critical for the morphogenesis of stereocilia and auditory perception by restricting taperin to the stereocilia base.

## In Brief

Liu et al. show that GRXCR2 and taperin form a complex at the base of the stereocilia in cochlear hair cells. Stereocilia lacking GRXCR2 are longer than normal and disorganized due to the mislocalization of taperin, which could modulate the actin cytoskeleton in stereocilia. Reducing taperin expression levels could rescue the morphological defects of stereocilia and restore the hearing of *Grxcr2*-deficient mice.

## Graphical Abstract

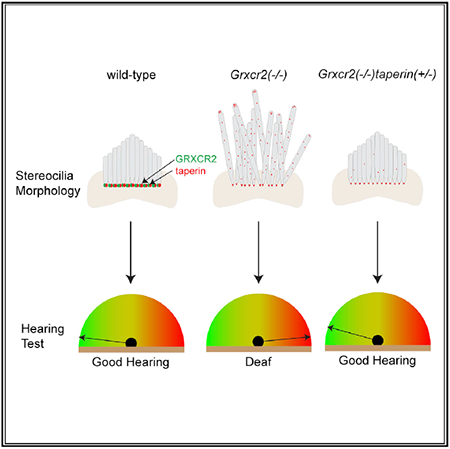

## INTRODUCTION

**Figure 1.**
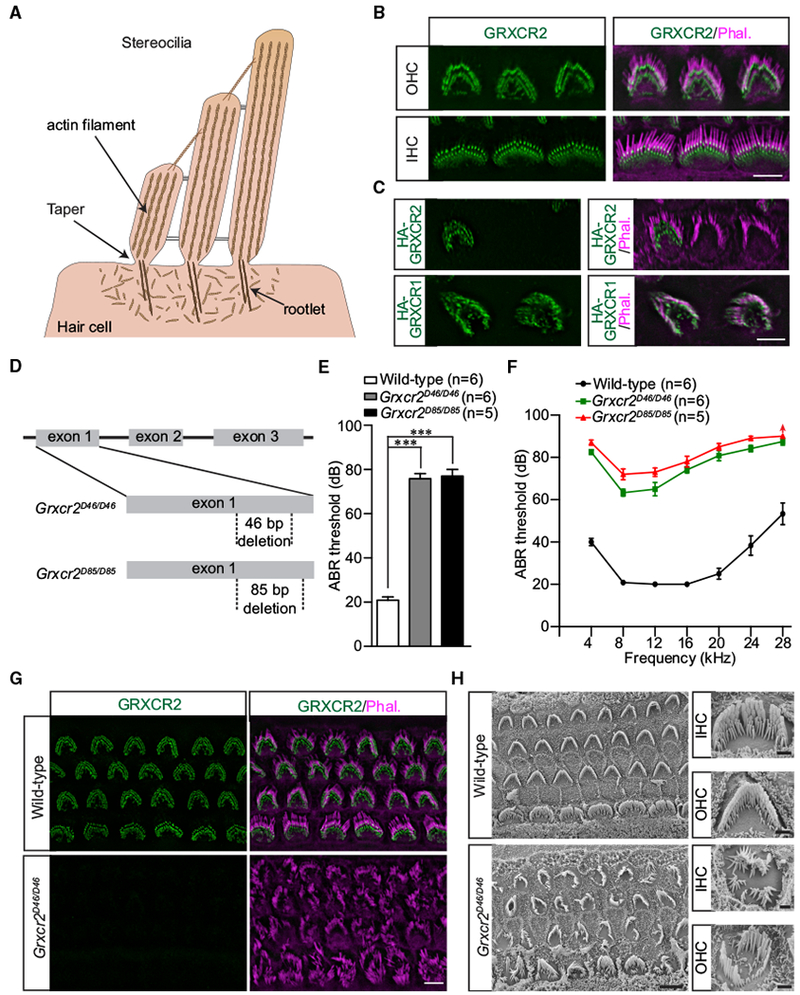
*Grxcr2*-Deficient Mice Are Deaf with Disorganized Stereocilia (A) Diagram of cochlear hair cell showing the mechanically sensitive stereocilia of graded height. Stereocilia contain hundreds of tightly cross-linked and uniformly polarized actin filaments, which provide the stiffness of stereocilia. At the base, stereocilia form a taper. The diagram is modified from Zhao et al. (2016). (B) Costaining of P7 cochlear whole mounts with GRXCR2-antibody (green) and phalloidin (magenta) to reveal stereocilia. Note the localization of GRXCR2 at the base of the stereocilia in both inner hair cells (IHCs) and outer hair cells (OHCs). Scale bar: 5 μm. (C) Cochlear explants were prepared at P3 and injectoporated to express HA-GRXCR2 and HA-GRXCR1, respectively. Two days later, the cells were fixed and stained with the HA-antibody. Note HA-GRXCR2 was localized at the base of the stereocilia, while HA-GRXCR1 was localized throughout the stereocilia. Scale bar: 5 μm. (D) Diagram of the strategy to generate *Grxcr2*-deficient mice. sgRNAs targeting exon 1 of *Grxcr2* induced 46-bp and 85-bp nucleotides deletions, respectively. (E and F) ABR thresholds for click stimuli (E) and pure tones (F) at 5 weeks of age. Note that the highest sound intensity tested was 90 dB. *Grxcr2^D85/D85^* mice had no response to the 90-dB pure tone stimuli at 28 kHz, and the threshold at this point was assigned as 90 dB. The number of analyzed mice is indicated in the brackets. The results are represented as the mean ± SEM. ***p < 0.001 by Student’s t test. (G) Cochlear whole mounts from wild-type and *Grxcr2^D46/D46^* mice at P5 were stained for GRXCR2 and phalloid in to reveal stereocilia. Note the absence of a signal in the mutant mice with disorganized stereocilia. Scale bar: 5 μm. (H) Scanning electron microscope images showing auditory sensory epithelia of wild-type (upper panel) and *Grxcr2^D46/D46^* (lower panel) mice at the age of P7. Note that the stereocilia of IHCs and OHCs from mutants lacking GRXCR2 were disorganized. Scale bars: left panel, 5 μm; right panel, 1 μm.

Human sensorineural hearing loss, the most common form of deafness, is often caused by defects in stereocilia (Barr-Gillespie, 2015; Richardson et al., 2011). Found on sensory hair cells in the inner ear, stereocilia are the mechanically sensitive, actin-based protrusions responsible for converting force from sound waves into electrical signals (Fettiplace and Kim, 2014; Gillespie and Müller, 2009; Hudspeth, 2008). This function depends on the organization and morphology of stereocilia. On each hair cell, several stereocilia form a bundle organized into rows of decreasing height. Sound displaces the bundle toward the tallest row, requiring the stereocilia to bend. Stereocilia are stiff due to a core of tightly cross-linked actin filaments, but they can flex at the base where the structure narrows in a taper region that is anchored by rootlet filaments (Figure 1A) (Barr-Gillespie, 2015).

In addition to providing a pivot point, the stereocilia base is organized by proteins linked to human deafness that are necessary for stereocilia formation and maintenance. Usher deaf-blindness syndrome proteins USH2A and VLGR1 together with scaffolding proteins Whirlin and PDZD7, which binds MYO7A, form ankle links between adjacent stereocilia required for bundle development (Chen et al., 2014; Grati et al., 2012; Michalski et al., 2007; Morgan et al., 2016). TRIOBP organizes actin filaments to form the rootlet, which provides durability and rigidity for the mechanosensitivity of stereocilia (Kitajiri et al., 2010). CLIC5 stabilizes membrane-actin filament linkages by forming a complex with radixin, taperin, MYO6, and PTPRQ at the base of the stereocilia (Salles et al., 2014). Taperin, mutations of which lead to deafness in humans (Li et al., 2010; Rehman et al., 2010), forms a dense-core-like structure encircled by an oligomeric ring of Fam65b at the taper region of stereocilia (Zhao et al., 2016). However, the function of taperin in stereocilia is unknown.

GRXCR2 and its paralog GRXCR1 are ~30 kDa in size and are highly conserved cytosolic proteins. Mutations in GRXCR2 and GRXCR1 have been linked to hearing loss in humans (Imtiaz et al., 2014; Odeh et al., 2010; Schraders et al., 2010a), but the underlying mechanisms are still unknown. Mutant mice lacking *Grxcr2* and *Grxcr1* have profound hearing loss and disorganized stereocilia (Avenarius, 2012; Odeh et al., 2010). Previous studies found that GRXCR1 is localized throughout the length of stereocilia (Odeh et al., 2010). In contrast, we found that GRXCR2 is concentrated at the basal region of stereocilia and is critical for the localization of taperin. In *Grxcr2*-deficient hair cells, taperin is reduced at the base and instead localizes along the length of the stereocilia, which are elongated and disorganized. A similar stereocilia phenotype also resulted from overexpression of taperin. In addition, ectopic taperin expression in COS7 cells caused actin rod formation. Together, these data suggest that mislocalized taperin activity in *Grxcr2*-deficient mice might be a cause of stereocilia defects and hearing loss. Consistent with this hypothesis, reducing taperin expression restored stereocilia morphology and hearing in *Grxcr2*-deficient mice. Thus, our findings demonstrate that GRXCR2 restricts taperin to the stereocilia base, and that mislocalized taperin activity disrupts stereocilia morphogenesis to cause deafness.

## RESULTS

### GRXCR2 Is Concentrated at the Base of the Stereocilia, and *Grxcr2*-Deficient Mice Are Deaf

*GRXCR2* mutation has been linked to hearing loss in humans (Imtiaz et al., 2014). Using a commercially available antibody (Sigma), we analyzed its expression pattern in the inner ear by immunohistochemistry. In whole mounts of the cochlea at postnatal day 7 (P7), we detected GRXCR2 immunoreactivity at the basal end of the stereocilia near the taper region in both outer hair cells (OHCs) and inner hair cells (IHCs) (Figure 1B). To further define the localization of GRXCR2, we stained GRXCR2 in the inner ear cryosections and found that GRXCR2 was concentrated at the basal taper region of the stereocilia (Figure S1A). Moreover, we used injectoporation (Xiong et al., 2014) to express hemagglutinin (HA)-tagged GRXCR2 in hair cells at P3. We fixed and stained the injectoporated hair cells 2 days later with HA-antibody to detect HA-GRXCR2. Consistent with the immunolocalization data using the GRXCR2-antibody, HA-GRXCR2 was concentrated near the base of the stereocilia (Figure 1C). In the adult hair cells, GRXCR2 was still concentrated at the basal taper region, but some immunostaining signals of GRXCR2 were also detected in the stereocilia shaft (Figure S1B). GRXCR1 was previously reported to localize throughout the length of stereocilia (Odeh et al., 2010). Correspondingly, we found that injectoporated HA-GRXCR1 was distributed throughout stereocilia (Figure 1C). Thus, GRXCR1 and GRXCR2 have distinct localization patterns in stereocilia. Notably, no obvious changes in stereocilia were observed in the hair cells injectoporated with either HA-GRXCR2 or HA-GRXCR1 (Figure 1C).

To gain insight into the mechanisms by which mutations in *Grxcr2* cause hearing loss, we used the clustered regularly interspaced short palindromic repeats (CRISPR)/Cas9 system to introduce mutations in exon 1 of *Grxcr2*. Two new alleles were generated, bearing 46-bp (*Grxcr2^D46/D46^*) and 85-bp (*Grxcr2^D85/D85^*) deletions in exon 1. The frameshifts caused by both deletions in the DNA sequence created early stop codons at different sites (Figure 1D). To characterize the auditory function of these mice, we measured the auditory brainstem response (ABR) to broadband click stimuli in 5-week-old animals. Neither *Grxcr2^D46/D46^* nor *Grxcr2^D85/D85^* mice could respond to the ~80-dB sound stimuli, suggesting that they were profoundly deaf (Figure 1E). Recording ABRs in response to pure tones revealed that both of these mice had hearing loss across the entire analyzed frequency spectrum (Figure 1F). Hearing loss likely results from loss of GRXCR2 function since immunoreactivity against the protein was completely abolished in *Grxcr2^D46/D46^* and *Grxcr2^D85/D85^* hair cells (Figures 1G and S1C). These results show that GRXCR2 is required for hearing.

### *Grxcr2*-Deficient Mice Show Disorganized Stereocilia Bundles

Since *Grxcr2* mutations cause deafness, we characterized hair bundle morphology in *Grxcr2^D46/D46^* and *Grxcr2^D85/D85^* hair cells by staining whole-mount cochlea with phalloidin to detect F-actin in stereocilia. At P2, some IHCs had disorganized stereocilia and OHCs showed defects in hair bundle polarity (Figure S1D). At P5, most of the OHCs and IHCs had severely disorganized stereocilia (Figures 1G and S1C).

To analyze the structural defects in stereocilia in more detail, we carried out scanning electron microscopy analysis of hair cells at P7. In wild-type mice, both IHC and OHC stereocilia bundles were uniformly oriented and showed a staircase-like organization with rows of stereocilia of graded height (Figure 1H). In contrast, most of the IHC and OHC bundles in *Grxcr2*-deficient mice were disorganized and had lost their characteristic V shapes. Some mutant bundles were rounded or fragmented with signs of degeneration, indicating that bundle cohesion was also affected (Figure 1H).

### GRXCR2 Interacts with Taperin

**Figure 2.**
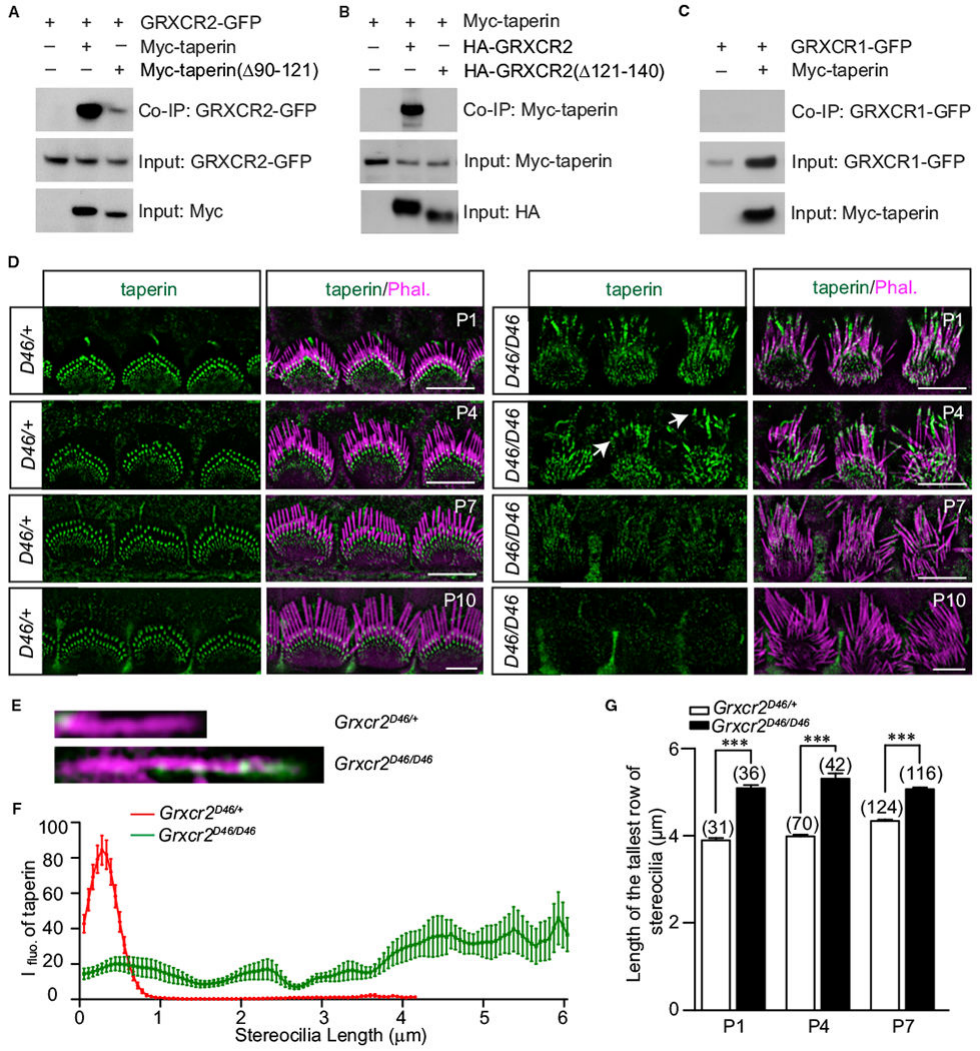
GRXCR2 Interacts with Taperin and Is Critical for the Localization of Taperin to the Basal Stereocilia (A–C) HEK293 cells were transfected with the constructs indicated on the top of each panel. Immunoprecipitations were carried out with Myc-antibody (A and C) or HA-antibody (B) followed by western blotting to detect co expressed proteins. The upper rows show co-IP results, and the lower rows show input proteins. (D) Cochlear whole mounts at P1–P10 were stained for taperin and phalloidin. Taperin was localized at the base of the stereocilia in control IHCs at different developmental stages. Notably, taperin staining was diffused at P1–P4 *Grxcr2^D46/D46^* IHCs and some taperin was concentrated at the distal end of the stereocilia (arrows). Taperin staining was dramatically reduced in P7 and P10 hair cells without a significant change in supporting cells, which surround the hair cells. Scale bars: 5 μm. (E-G) Illustrated by taperin (green) and phalloid in (magenta) staining, single stereocilium from the tallest row could be selected (E), the fluorescence intensity of taperin staining (F), and the length of stereocilium (G) from the basal region (left) to the distal end (right) of stereocilia were measured by ImageJ (NIH). (F) Taperin staining in ten stereocilia per group from the tallest row of IHCs (n = 10) at the age of P1 was measured. In control stereocilia, taperin was concentrated at the base; however, in the *Grxcr2*-deficient hair cells, taperin was redistributed along the shaft of the stereocilia. (G) Approximately three to four stereocilia of the tallest row per IHC were measured, and the number of analyzed IHCs is indicated in brackets. All values are represented as the mean ± SEM. ***p < 0.001 by Student’s t test.

Taperin, mutations of which have been linked to hearing loss in humans and mice, is a cytosolic protein with 749 aa (Chen et al., 2016; Li et al., 2010; Rehman et al., 2010). Within hair cells, taperin localizes to the basal region of cochlear hair cell stereocilia (Rehman et al., 2010; Zhao et al., 2016) (Figures S2A–S2C), putting it in proximity to GRXCR2. To test whether there is interaction between GRXCR2 and taperin, we carried out co-immunoprecipitation (coIP) experiments with extracts from HEK293 cells that were transfected with Myc-tagged taperin and GFP-tagged GRXCR2. GFP-GRXCR2 could be co-immunoprecipitated with Myc-taperin (Figure 2A). Correspondingly, Myc-taperin could be co-immunoprecipitated with HA-GRXCR2 (Figure 2B). To identify regions in taperin and GRXCR2 critical for interaction, we next generated several truncated taperin and GRXCR2 constructs and their interactions were tested (Figures S2D–S2G). Finally, we found that the 30 aa from 90–121 in taperin and 20 aa from 121–140 in GRXCR2 are important for their interactions (Figures 2A and 2B). In contrast to GRXCR2, GRXCR1-GFP could not be co-immunoprecipitated with Myc-taperin (Figure 2C). Different binding partners and different localization patterns suggest that GRXCR2 and GRXCR1 act, at least in part, differently in cochlear hair cells.

### Expression of Taperin in *Grxcr2*-Deficient Hair Cells

Our immunolocalization and biochemical data suggest that GRXCR2 and taperin may form a complex at the base of the stereocilia. To investigate the localization of taperin in *Grxcr2*-deficient hair cells, immunohistochemistry using a commercially available antibody against taperin (Sigma) was performed. In the *Grxcr2*^*D46/*+^ hair cells, taperin was concentrated at the basal taper region of the stereocilia at different developmental stages (Figures 2D and 2F). However, in P1 and P4 *Grxcr2*-deficient hair cells, taperin staining was typically diffused along the stereocilia length and sometimes accumulated toward the distal end of the stereocilia (Figures 2D–2F). By staining taperin and phalloidin (Figure 2E), we were able to measure the length of the tallest row of stereocilia in wild-type and *Grxcr2*-deficient hair cells that was revealed (Figure 2G). Remarkably, stereocilia of *Grxcr2*-deficient hair cells were longer than wild-type hair cells (Figures 2D, 2G, and S2H). By P7 and P10, the expression of taperin was dramatically reduced in the *Grxcr2*-deficient hair cells (Figure 2D), which may be due to the degradation of the mislocalized taperin or degeneration of stereocilia. Notably, we did not observe a significant change in taperin staining in the supporting cells, which surround the hair cells (Figure 2D). These results demonstrate that GRXCR2 is important for the restriction of taperin to the basal region of stereocilia in hair cells.

### *Taperin*-Deficient Mice Are Deaf

**Figure 3.**
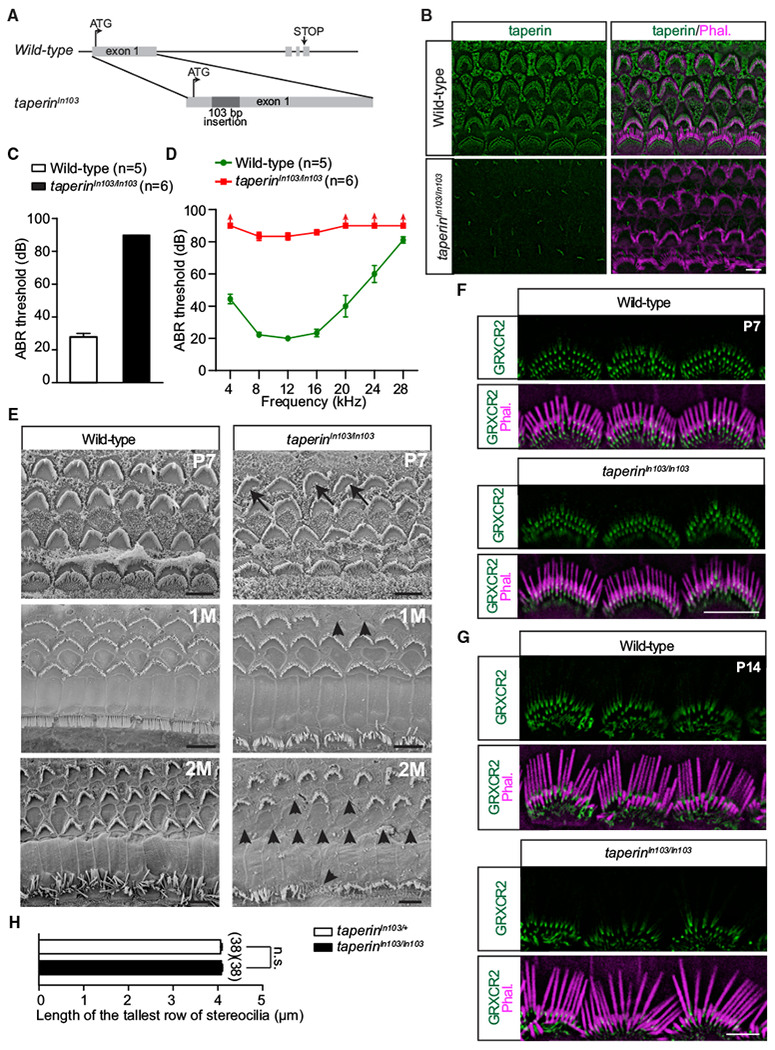
*Taperin*-Deficient Mice Are Deaf (A) Diagram of the strategy to generate *taperin*-deficient mice by using the CRISPR/Cas9 system. sgRNAs targeting exon 1 of *taperin* induced a 103-bp nucleotide insertion, which generated an early stop codon. (B) Cochlear whole mounts from wild-type mice and *taperin^In103/In103^* mice at P5 were stained for taperin (green) and phalloidin (magenta) to indicate the stereocilia. Note that there is no staining at the base of the stereocilia or in supporting cells in taperin^In103/In103^ mice. The weak residual immunoreactivity in *taperin^In103/In103^* tissue is likely caused by nonspecific antibody binding. (C and D) ABR thresholds for click stimuli (C) and pure tones (D) at 2 months of age. Note that the highest sound intensity tested was 90 dB. *taperin^In103/In103^* mice had no response to the 90-dB broadband click stimuli or pure tones at 4, 20, 24, and 28 kHz, and the threshold was assigned as 90 dB. The number of analyzed mice is indicated in the brackets. The results are represented as mean ± SEM. (E) Scanning electron microscope images showing auditory sensory epithelia of wild-type (left panel) and *taperin^In103/In103^* (right panel) mice at P7 and 1 and 2 months of age. Note, some hair cells from *taperin^In103/In103^* mice had asymmetric V-shaped bundles at P7 (arrows). Note hair cell loss (arrowheads) in mutant mice at 1 and 2 months of age. (F and G) GRXCR2 staining in control and mutant IHCs at P7 (F) and P14 (G). Stereocilia were visualized by staining with phalloidin (magenta). Note that GRXCR2 staining showed no significant change in mutant mice. (H) Length of the tallest row of stereocilia of IHCs. Cochlear whole mounts from P4 control and *taperin^In103/In103^* animals were stained for GRXCR2 and phalloidin. The length of the tallest row of stereocilia was measured with ImageJ by measuring the distance from the distal end to the basal region of the stereocilia, which were revealed by phalloidin and GRXCR2 staining. Approximately three to four stereocilia of the tallest row per cell were measured. The number of analyzed IHCs is indicated in brackets. All values are mean ± SEM. n.s., not significant by the Student’s t test. Scale bars: 5 μm.

To investigate the function of taperin in hearing and GRXCR2 function, we generated *taperin*-deficient mice using the CRISPR/Cas9 system. *Taperin* has four exons (Figure 3A) and all reported mutations linked to human deafness are located in exon 1 (Bashir et al., 2013; Li et al., 2010; Rehman et al., 2010; Wang et al., 2014), suggesting it encodes a sequence critical for taperin function. Thus, single guide RNAs (sgRNAs) were designed to target exon 1 of *taperin*. Finally, we obtained a mouse line with a 103-bp nucleotide insertion between nucleotides 217 and 218 in exon 1 of *taperin* (*taperin^In103/In103^*). The frameshift caused by the 103-bp insertion created an early stop codon directly stopping protein translation (Figure 3A). Immunostaining was then performed to test whether taperin was depleted in the *taperin^In103/In103^* mice. In wild-type mice, taperin was localized at the base of the stereocilia, as well as in supporting cells (Figure 3B). Immunostaining of stereocilia and support cells was completely abolished in *taperin^In103/In103^* mice. Some weak residual immunoreactivity mainly at the kinocillium-like structure was likely caused by nonspecific antibody binding (Figure 3B). To characterize the auditory function of *taperin^In103/In103^* mice, we measured ABRs to broadband click or pure tone stimuli in 2-month-old animals and found that *taperin^In103/In103^* mice had profound hearing loss (Figures 3C and 3D), demonstrating that taperin is essential for hearing.

### Stereocilia Morphology and GRXCR2 Expression in *Taperin*-Deficient Hair Cells

To analyze the morphology of *taperin*-deficient stereocilia, we imaged hair cells from *taperin^In103/In103^* mice using both scanning and transmission electron microscopy. At P7, most hair cells from the *taperin^In103/In103^* mouse appeared to be fairly normal by scanning electron microscopy, with only a few cells displaying asymmetric V-shaped bundles (Figure 3E). Similarly, we did not detect any defects in the rootlet using transmission electron microscope (Figure S3A). Despite apparently normal development, by 1 month of age there was occasional hair cell loss and by 2 months of age there was significant hair cell loss in the *taperin^In103/In103^* inner ear (Figure 3E).

As the localization of taperin was altered in the *Grxcr2*-deficient hair cells, we also analyzed the localization of GRXCR2 in the *taperin^In103/In103^* mouse. However, GRXCR2 immunostaining was similar in *taperin^In103/In103^* and wild-type hair cells (Figures 3F, 3G, and S3B), suggesting that taperin is not required for the GRXCR2 localization during development. Revealed by GRXCR2 and phalloidin staining, we were able to measure the length of the tallest row of stereocilia in wild-type and *taperin*-deficient IHCs. At the age of P4, the length of stereocilia of *taperin*-deficient hair cells had no significant change compared with *taperin*^*In103/*+^ hair cells (Figure 3H).

### Taperin Can Modulate the Actin Cytoskeleton

**Figure 4.**
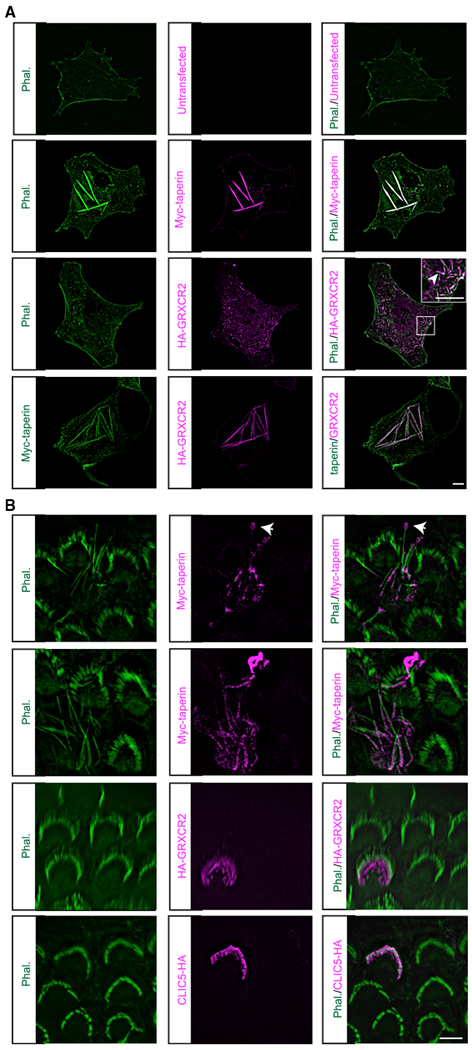
Taperin Can Modulate the Actin Cytoskeleton in COS7 Cells and Hair Cells (A) COS7 cells were transfected to express Myc-taperin and/or HA-GRXCR2. Note rod-like structures (arrows)formed by actin filaments and Myc-taperin in transfected COS7 cells. HA-GRXCR2 partially co localized with actin filament (arrowhead) but could not regulate the actin cytoskeleton in COS7 cells. HA-GRXCR2 and Myc-taperin co localized in the rod-like structure. Scale bar: 10 μm. (B) Injectoporation of Myc-taperin but not HA-GRXCR2 or CLIC5-HA led to the extraordinary growth of stereocilia. Note that Myc-taperin was diffused in the stereocilia. Some Myc-taperin was concentrated at the distal end of the stereocilia (arrow). Scale bar: 5 μm.

Taperin shows an overall 34% similarity to phostensin (Rehman et al., 2010), which can cap the pointed end of actin filaments and modulate actin dynamics (Kao et al., 2007; Lai et al., 2009). To assess the potential of taperin to regulate actin structures, we overexpressed Myc-taperin in COS7 cells. Rod-like aggregates containing actin and Myc-taperin were formed in ~53% of transfected COS7 cells (Figure 4A). In contrast, overexpression of GRXCR2, which partially colocalized with F-actin, had only minor effects on the actin structure in COS7 cells (Figure 4A). In COS7 cells transfected with Myc-taperin and HA-GRXCR2, Myc-taperin and HA-GRXCR2 colocalized in the rod-like F-actin structure (Figure 4A; data not shown).

To investigate whether taperin could regulate the actin cytoskeleton in cochlear hair cells, we expressed Myc-taperin in hair cells at P3 using injectoporation. The hair cells were fixed 2 days after injectoporation and stained with phalloidin to reveal stereocilia and with Myc-antibody to detect Myc-taperin. Interestingly, we found that the length of stereocilia was much longer in the OHCs expressing taperin (8.45 ± 0.68 μm, n = 10), compared to neighboring untransfected OHCs (3.02 ± 0.06 μm, n = 13). More than half of the hair cells injectoporated with Myc-taperin had severely disorganized stereocilia, while injectoporation of HA-GRXCR2 or CLIC5-HA, another protein localizing at the base of the stereocilia in hair cells (Gagnon et al., 2006; Salles et al., 2014), did not alter the morphology of stereocilia (Figure 4B). Injectoporation of untagged taperin also led to the extraordinary growth of stereocilia, excluding the possibility that the over-elongation of stereocilia was caused by Myc-tag-induced aberrant protein activity (Figure S4A). The 30 aa from 90–121 in taperin are important for the interaction with GRXCR2 (Figure 2A). We injectoporated Myc-taperin(Δ90–121) in hair cells and found that the injectoporated hair cells had longer stereocilia, suggesting that the GRXCR2 binding region of taperin is not critical for modulating the actin cytoskeleton in hair cells (Figure S4B). Murine taperin has 749 aa, encoded by four exons (Figure 3A). Exon 1 encodes the first 622 aa. So far, all reported mutations linked to human deafness are located in exon 1 (Bashir et al., 2013; Li et al., 2010; Rehman et al., 2010; Wang et al., 2014), suggesting it encodes a sequence critical for taperin function. We used injectoporation to express HA-taperin(1–622) in hair cells. The injectoporation of HA-taperin(1–622) also led to the extraordinary growth of stereocilia, although the effects were not as strong as the injectoporation of full-length taperin (Figure S4B). A human *taperin* splicing isoform, missing the first 306 aa, has been reported (Gerhard et al., 2004; Ota et al., 2004). As the murine and human taperin proteins are highly homologous, we constructed a plasmid expressing a taperin protein missing the first 346 aa, corresponding to the human splicing isoform. Interestingly, HA-taperin(347–end) did not localize to the stereocilia nor induce the extraordinary growth of stereocilia (Figure S4B).

While endogenous taperin localized to the stereocilia base, overexpressed Myc-taperin was found along the stereocilia length, with some protein accumulated toward the distal end of the stereocilia (Figure 4B). The mislocalization of over expressed taperin might have been caused by the limited amount of endogenous scaffold proteins, such as GRXCR2, at the basal stereocilia. Notably, taperin was similarly mislocalized in *Grxcr2*-deficient hair cell stereocilia, which were also disorganized and longer than normal (Figures 2D–2G and S2H). Together, these data suggest that GRXCR2 is critical for the morphology of stereocilia possibly by preventing taperin from inappropriately accessing to the stereocilia shaft.

### Reducing Taperin Expression Rescued the Morphological Defects Caused by Loss of GRXCR2

**Figure 5.**
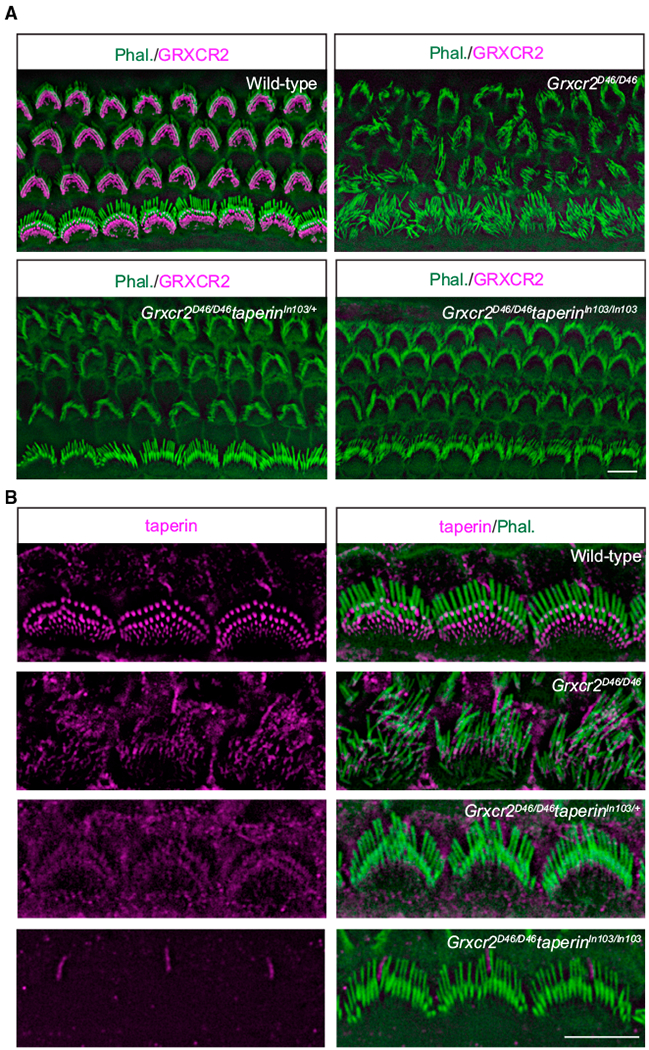
Analysis of Hair Bundle Morphology by Whole-Mount Staining (A) Whole mounts from the middle part of the cochlea from wild-type, *Grxcr2^D46/D46^, Grxcr2^D46/D46^taperin*^*In103/*+^, and *Grxcr2^D46/D46^taperin^In103/In103^* mice at P7 were stained with an antibody to GRXCR2. Stereocilia were identified by phalloidin (green) counterstaining. Note that hair cells from *Grxcr2^D46/D46^* mouse had disorganized stereocilia. Remarkably, stereocilia from *Grxcr2^D46/D46^taperin*^*In103/*+^ and *Grxcr2^D46/D46^taperin^In103/In103^* mice were well maintained. (B) IHCs from wild-type, *Grxcr2^D46/D46^, Grxcr2^D46/D46^taperin*^*In103/*+^, and *Grxcr2^D46/D46^taperin^In103/In103^* mice at P7 were stained with an antibody to taperin. Note that there is no taperin staining at the base of the stereocilia or in supporting cells in *Grxcr2^D46/D46^taperin^In103/In103^* mice. The weak residual immunoreactivity in *Grxcr2^D46/D46^taperin^In103/In103^* tissue is likely caused by nonspecific antibody binding. Stereocilia were identified by phalloidin (green) counter-staining. Scale bars: 5 μm.

To investigate whether the disorganization of stereocilia in *Grxcr2*-deficient hair cells is caused by taperin, we crossed *Grxcr2^D46/D46^* mice with *taperin^In103/In103^* mice. We then characterized the hair bundle morphology in *Grxcr2^D46/D46^* mice with different *taperin* genotypes. In the *Grxcr2^D46/D46^taperin*^+/+^ hair cells, the stereocilia were disorganized. In contrast, most hair cells from *Grxcr2^D46/D46^taperin*^*In103/*+^ and *Grxcr2^D46/D46^taperin^In103/In103^* mice were phenotypically normal with a nicely arranged V-shaped hair bundle at P7 (Figures 5A and 5B). Immunostaining revealed that taperin was dramatically reduced in the *Grxcr2^D46/D46^taperin*^*In103/*+^ hair cells and abolished in the *Grxcr2^D46/D46^taperin^In103/In103^* hair cells (Figure 5B).

**Figure 6.**
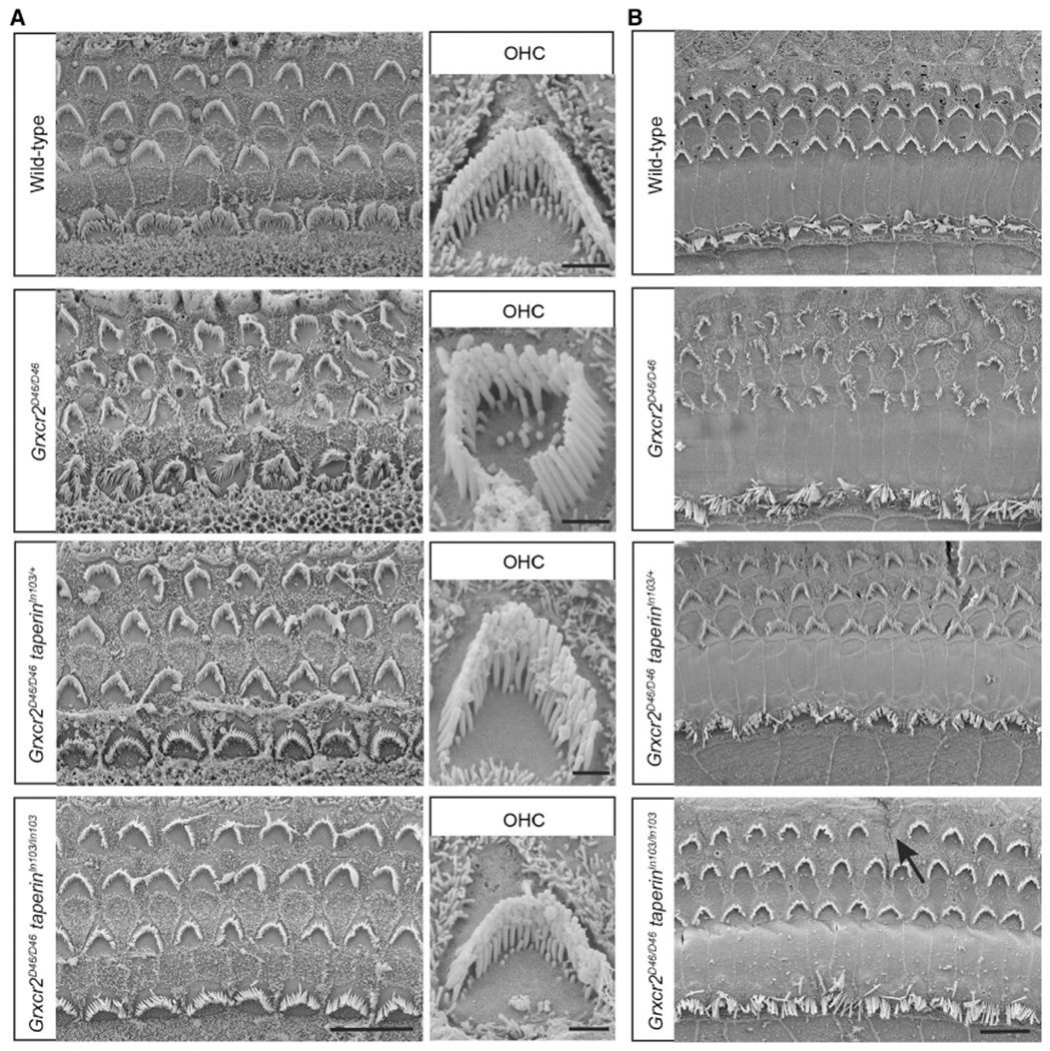
Analysis of Hair Bundle Morphology by Scanning Electron Microscopy (A and B) Whole mounts from the middle part of the cochlea from wild-type, *Grxcr2^D46/D46^, Grxcr2^D46/D46^taperin*^*In103/*+^, and *Grxcr2^D46/D46^taperin^In103/In103^* mice at the age of P7 (A) and 1 month (B) were analyzed by scanning electron microscopy. Most of the IHCs and OHCs in *Grxcr2^D46/D46^* cochlea showed disorganization of hair bundles, reflected in a loss of the V-shaped hair bundle. Remarkably, most of the hair cells in *Grxcr2^D46/D46^taperin*^*In103/*+^ and *Grxcr2^D46/D46^taperin^In103/In103^* cochlea had a classical V-shaped hair bundle. Hair cell loss (arrow) could be found in the *Grxcr2^D46/D46^taperin^In103/In103^* cochlea at the age of 1 month, which is probably caused by *taperin* depletion. Scale bars: (A) left panel, 10 μm; right panel, 1 μm; (B) 10 μm.

To analyze the structure of stereocilia in more detail, we carried out additional scanning electron microscopy analyses with hair cells at P7 and 1 month of age. In the *Grxcr2^D46/D46^* mice, the stereocilia were disorganized (Figure 6). Most cochlear hair cells in the *Grxcr2^D46/D46^taperin*^*In103/*+^ mouse, having one *taperin* allele depleted in the genome, had a fairly normal V-shaped bundle. Only ~15% of P7 *Grxcr2^D46/D46^taperin*^*In103/*+^ hair cells, mainly OHCs, had observable morphological defects (Figure 6A). Morphology was corrected to an even greater extent in *Grxcr2^D46/D46^taperin^In103/In103^* mice, where more than 95% of hair cells had a standard V-shaped bundle (Figure 6A). By 1 month of age, most *Grxcr2^D46/D46^taperin*^*In103/*+^ hair cells had a classical V-shaped hair bundle, while occasional hair cell loss occurred in *Grxcr2^D46/D46^taperin^In103/In103^* mice (Figure 6B). These results demonstrate that stereocilia defects in the *Grxcr2* mutant are caused by the mislocalization of taperin because reducing taperin expression rescued stereocilia morphology.

### Reducing Taperin Expression Restored Auditory Function in *Grxcr2*-Deficient Mice

**Figure 7.**
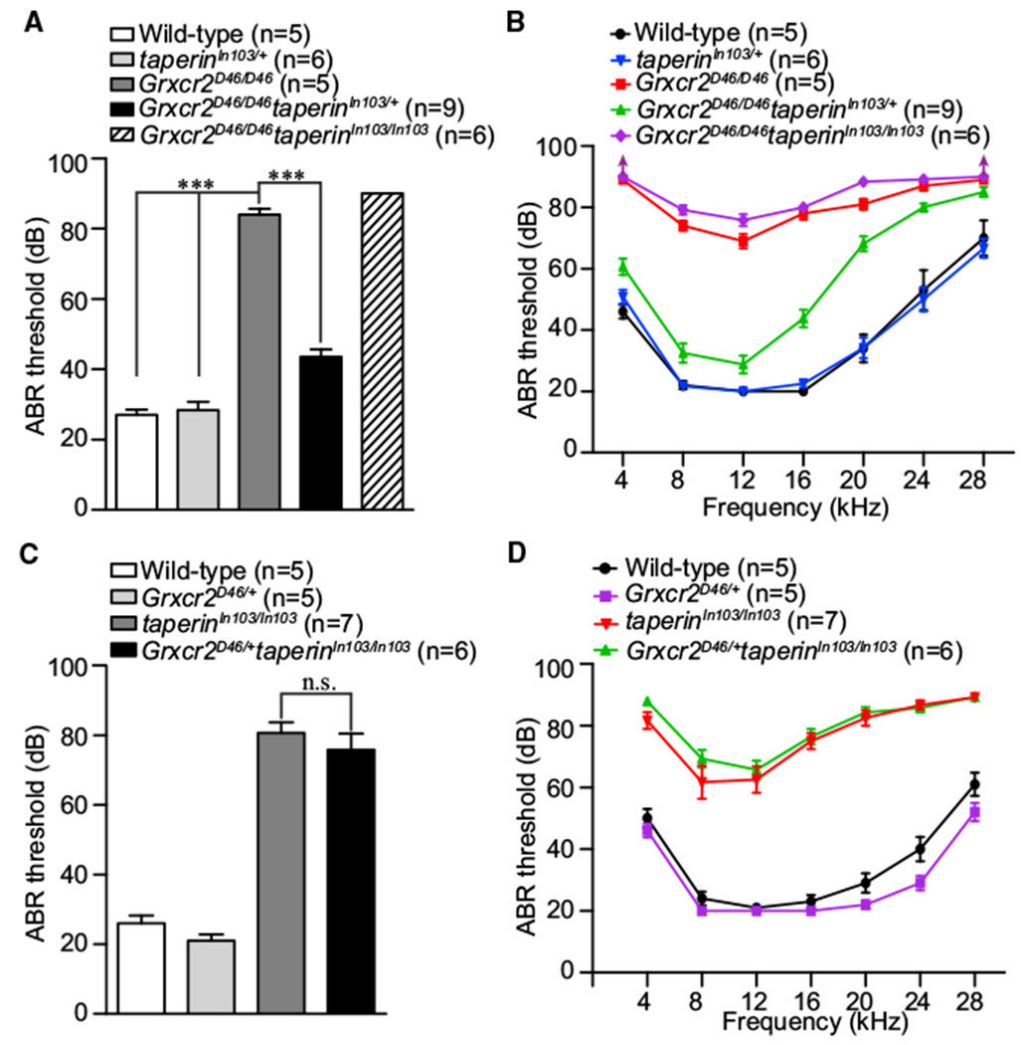
Analysis of Hearing Function by ABR (A–D) ABR thresholds for click stimuli (A and C) and pure tones (B and D) at 6 weeks of age. Note that the *Grxcr2^D46/D46^taperin*^*In103/*+^ mice, with one allele of *taperin* expression, had better hearing than *Grxcr2^D46/D46^* mice (A and B). The *Grxcr2^D46/D46^taperin^In103/In103^* mice (A and B) and *Grxcr2*^*D46/*+^*taperin^In103/In103^* mice (C and D) had profound hearing loss. *Grxcr2^D46/D46^taperin^In103/In103^* mice had no response to the 90-dB broadband click stimuli or pure tones at 4 and 28 kHz, and the threshold was assigned as 90 dB. The number of analyzed mice is indicated in brackets. Results are represented as the mean ± SEM. ***p < 0.001; n.s., not significant by Student’s t test.

To investigate whether inhibiting taperin expression, which rescued stereocilia morphology, could restore auditory function, we measured ABRs of 6-week-old *Grxcr2^D46/D46^taperin*^*In103/*+^ and *Grxcr2^D46/D46^taperin^In103/In103^* mice. Although *Grxcr2^D46/D46^taperin^In103/In103^* mice have fairly normal V-shaped stereocilia bundles (Figures 5 and 6), they still had profound hearing loss (Figures 7A and 7B). Remarkably, *Grxcr2^D46/D46^taperin*^*In103/*+^ mice, which lack only one allele of *taperin*, showed dramatically improved hearing compared to *Grxcr2^D46/D46^* mice (Figures 7A and 7B). The ABR threshold for a click stimulus was reduced to ~43-dB sound pressure level (SPL), and pure tone thresholds were also significantly lower, particularly at lower frequencies (Figures 7A and 7B). These results show that partially inhibiting taperin expression could restore hearing loss caused by loss of GRXCR2.

To investigate whether reducing GRXCR2 expression rescues hearing loss in *taperin^In103/In103^* mice, we also measured the auditory thresholds of *Grxcr2*^*D46/*+^*taperin^In103/In103^* mice. ABR thresholds were not significantly different between *taperin^In103/In103^* and *Grxcr2*^*D46/*+^*taperin^In103/In103^* mice (Figures 7C and 7D). Thus, these results suggest that taperin acts downstream of GRXCR2 in cochlear hair cells.

## DISCUSSION

The base of stereocilia exhibits a striking structural organization and is critical for hair cell function. We reveal that GRXCR2, a protein linked to hearing loss in humans (Imtiaz et al., 2014), is concentrated at this region, binds taperin, and restricts taperin localization to the stereocilia base. Inhibiting taperin expression rescued the morphological defects and hearing loss caused by the *Grxcr2* mutation. This mechanism shows that disrupting protein complexes at the stereocilia base causes stereocilia defects and hearing loss through mislocalized taperin activity.

We found that depleting one allele of *taperin* in the genome restored hearing loss caused by GRXCR2 mutation. Interestingly, the degree of rescue varied in *Grxcr2^D46/D46^taperin*^*In103/*+^ mice according to the frequency of sound; ABR thresholds for lower-pitched sounds were close to wild-type values but were significantly elevated for higher-pitched sounds (Figure 7B). Corresponding to the tonotopic organization of the cochlea, hair cells in the apical region detect low-frequency sound while those in the basal region detect high-frequency stimuli. The percentage of hair cells with normal stereociliary morphology at the apical region (85.7 ± 2.1%) was similar to the basal region (89.7 ± 1.3%) in *Grxcr2^D46/D46^taperin*^*In103/*+^ mice at the age of P7. Since high-frequency hearing was worse in *Grxcr2^D46/D46^taperin*^*In103/*+^ mice than in *taperin*^*In103/*+^ mice and some GRXCR2 entered the stereocilia shaft in adult wild-type hair cells (Figure S1B), GRXCR2 likely interacts with other proteins, which are probably not essential for stereocilia morphogenesis but required for high-frequency auditory perception.

Taperin has some similarity with phostensin, which caps F-actin pointed ends and regulates the elongation and depolymerization of actin filaments (Kao et al., 2007; Lai et al., 2009; Rehman et al., 2010). Consistently, taperin is localized to the basal taper region of the stereocilia, where many peripheral actin filaments with pointed ends terminate (Pataky et al., 2004; Rehman et al., 2010; Salles et al., 2014). In our studies, we found that overexpression of taperin in COS7 and HEK293 cells led to the formation of rod-like actin structures in more than 50% of transfected cells (Figure 4A; data not shown). Mislocalization of taperin along the stereocilia length, due to either overexpression or GRXCR2 loss, caused stereocilia elongation. This evidence suggests that taperin is an actin-cytoskeleton regulator and is probably involved in the regulation of actin dynamics at the pointed end in hair cells. To extensively illustrate the functions of taperin, further studies to evaluate its actin pointed-end capping ability and other possible functions on actin filament dynamics *in vitro* is important. Unfortunately, using bacterial expression systems, we could not purify full-length murine taperin to perform the *in vitro* biochemical experiments. Further studies to determine the functional domains of taperin and the extent to which these domains are involved in actin-cytoskeleton regulation would be informative.

Taperin is localized at the base of the stereocilia in hair cells of postnatal and adult mice (Rehman et al., 2010; Zhao et al., 2016). Loss of *taperin* leads to hearing loss in mice, which is consistent with mutations in *taperin* causing recessive hearing loss in humans and mice (Chen et al., 2016; Li et al., 2010; Rehman et al., 2010). The *taperin*-deficient mice progressively lost their hair cells (Figure 3E), which may have been caused by the instability of F-actin, inducing progressive degeneration of stereocilia. taperin is also expressed in the surrounding supporting cells (Rehman et al., 2010) (Figure 3B). Using the scanning electron microscope, we did not find any morphological defect in the supporting cells (Figure 3E). While our findings suggest that loss of hair cells was critical for the auditory phenotype caused by *taperin* knockout, depletion of *taperin* in the supporting cells might have also contributed to the auditory phenotype.

We found that GRXCR2 was concentrated at the base of the stereocilia and was critical for taperin localization. In the *taperin*-deficient hair cells, the localization of GRXCR2 showed no significant change (Figures 3F, 3G, and S3B), suggesting some other proteins at the base of the stereocilia might be important for the localization of GRXCR2. It will be of interest to investigate the underlying mechanisms that determine GRXCR2 localization. In addition to GRXCR2 and taperin, several proteins linked to deafness are concentrated at or near the stereocilia base, including Fam65b, PTPRQ, CLIC5, radixin, and PDZD7. Similar to GRXCR2, loss of these proteins leads to the morphological defects of stereocilia and hearing loss (Diaz-Horta et al., 2014; Khan et al., 2007; Morgan et al., 2016; Pataky et al., 2004; Salles et al., 2014; Schneider et al., 2009; Schraders et al., 2010b; Zhao et al., 2016). These phenotypes may also result from mislocalized taperin. Notably, taperin localization is altered in *Fam65b* and *Clic5* mutant hair cells (Salles et al., 2014; Zhao et al., 2016). In addition, radixin, PTPRQ, CLIC5, and MYO6 were shown to form a complex with taperin (Salles et al., 2014), which might also be required to localize taperin to the stereocilia base. We are currently systematically investigating the molecular interaction networks at the base of the stereocilia. The mechanism we present here, where GRXCR2 restricts taperin from entering the stereocilia shaft, may also explain the morphological defects and hearing loss caused by mutations of CLIC5, radixin, PTPRQ, MYO6, Fam65b, or other components located at the basal region of stereocilia. Thus, our studies provide leads linking several deafness-related proteins into a common molecular pathway and suggest that targeting taperin is an avenue for treating several forms of hearing loss.

## STAR★METHODS

### KEY RESOURCES TABLE

**Table:** 

REAGENT or RESOURCE	SOURCE	IDENTIFIER
Antibodies		
Anti-Taperin	Sigma	Cat# HPA020899; RRID: AB_1845835
Anti-GRXCR2	Sigma	Cat# HPA059421; RRID: AB_2684010
Anti-HA	Cell Signaling	Cat# 2367S; RRID: AB_10691311
Anti-Myc	Cell Signaling	Cat# 2278S; RRID: AB_10693332
Anti-Myc	Santa Cruz	Cat# sc-40; RRID: AB_627268
Anti-GFP	Santa Cruz	Cat# sc-9996; RRID: AB_627695
Goat anti-Rabbit IgG Secondary Antibody, Alexa Fluor 488	Invitrogen	Cat# A11070; RRID: AB_2534114
Goat anti-Mouse IgG Secondary Antibody, Alexa Fluor 488	Invitrogen	Cat# A11017; RRID: AB_2534084
Goat anti-Mouse IgG Secondary Antibody, Alexa Fluor 555	Invitrogen	Cat# A21425; RRID: AB_2535846
Goat anti-rabbit IgG Secondary Antibody, Alexa Fluor 546	Invitrogen	Cat# A11071; RRID: AB_2534115
Amersham ECL Rabbit IgG, HRP-linked whole Ab	GE Healthcare	Cat# NA934; RRID: AB_2722659
Amersham ECL Mouse IgG, HRP-linked whole Ab	GE Healthcare	Cat# NXA931; RRID: AB_772209
Bacterial and Virus Strains		
Subcloning Efficiency DH5α Competent Cells	Invitrogen	Cat# 18265017
MAX Efficiency Stbl2 Competent Cells	Invitrogen	Cat# 10268019
Chemicals, Peptides, and Recombinant Proteins		
Alexa Fluor 488 Phalloidin	Invitrogen	Cat# A12379
Alexa Fluor 568 phalloidin	Invitrogen	Cat# A12380
Alexa Fluor 647 phalloidin	Invitrogen	Cat# A22287
Thermo Scientific Pierce ECL 2 Western Blotting Substrate	Fisher Scientific	Cat# PI80196
32% Paraformaldehyde	Electron Microscopy Sciences	Cat# 15714
25% glutaraldehyde	Electron Microscopy Sciences	Cat# 16220
4% OsO_4_ solution	Electron Microscopy Sciences	Cat# 19150
Taq DNA Polymerase w/Thermo Pol Buffer	New England Biolabs	Cat# M0267X
Deoxynucleotide Solution	New England Biolabs	Cat# N0446S
Platinum Pfx DNA Polymerase	Invitrogen	Cat# 11708013
HBSS	Invitrogen	Cat# 14175103
DMEM	Invitrogen	Cat# 11965118
Penicillin-Streptomycin, liquid	Invitrogen	Cat# 15140122
Fetal bovine serum	Fisher Scientific	Cat# MT35010CV
EZview Red Anti-HA Affinity Gel	Sigma	E6779
EZview Red Anti-c-Myc Affinity Gel	Sigma	E6654
Experimental Models: Cell Lines		
HEK293	ATCC	Cat# CRL-1573
COS7	ATCC	Cat# CRL-1651
Experimental Models: Organisms/Strains		
Mouse: C57BL/6J	The Jackson Laboratory	Cat# 000664
Mouse: *Grxcr2^D46/D46^*	This study	n/a
Mouse: *Grxcr2^D85/D85^*	This study	n/a
Mouse: *taperin^In103/In103^*	This study	n/a
Mouse: *Grxcr2^D46/D46^taperin*^*In103/*+^	This study	n/a
Mouse: *Grxcr2^D46/D46^taperin^In103/In103^*	This study	n/a
Mouse: *Grxcr2*^*D46/*+^*taperin^In103/In103^*	This study	n/a
Oligonucleotides		
gRNA1 for generation of *Grxcr2^D46/D46^* and *Grxcr2^D85/D85^* mice: GGATGGCGTTTATGGGTCTG gttttagagctagaaatagcaagttaaaataaggctagtccgttatcaacttgaaaaagtggcaccgagtcggtgctttt	This study	n/a
gRNA2 for generation of *Grxcr2^D46/D46^* and *Grxcr2^D85/D85^* mice: GCAGCGGCGCCTACACTCTGG gttttagagctagaaatagcaagttaaaataaggctagtccgttatcaacttgaaaaagtggcaccgagtcggtgctttt	This study	n/a
gRNA1 for generation of *taperin^In103/In103^* mice: GCGACGGCACTGCCGGCCCCG gttttagagctagaaatagcaagttaaaataaggctagtccgttatcaacttgaaaaagtggcaccgagtcggtgctttt	This study	n/a
gRNA2 for generation of *taperin^In103/In103^* mice: GGATCTGGAGCGGCGCCGGA gttttagagctagaaatagcaagttaaaataaggctagt ccgttatcaacttgaaaaagtggcaccgagtcggtgctttt	This study	n/a
Genotyping primers for *Grxcr2^D46/D46^* and *Grxcr2^D85/D85^* mice (Forward): TCTTCCTACAGTGGCCGAGT	This study	n/a
Genotyping primers for *Grxcr2^D46/D46^* and *Grxcr2^D85/D85^* mice (Reverse): TGAATGTGAGCGAGATACCG	This study	n/a
Genotyping primers for *taperin^In103/In103^* mice (Forward): CTGGAAACGGGAGATCCTTG	This study	n/a
Genotyping primers for *taperin^In103/In103^* mice (Reverse): GAAGCCTGGCGCTGACTC	This study	n/a
Recombinant DNA		
Myc-taperin	Zhao et al., 2016	n/a
HA-GRXCR2	This study	n/a
HA-GRXCR1	This study	n/a
GRXCR2-GFP	This study	n/a
GRXCR1-GFP	This study	n/a
Myc-taperin(Δ90-121)	This study	n/a
HA-GRXCR2(Δ121-140)	This study	n/a
Software and Algorithms		
Prism 7	Graphpad	https://www.graphpad.com/
Vector NTI	Invitrogen	https://www.thermofisher.com/us/en/home/life-science/cloning/vector-nti-software.html
ImageJ	NIH	https://imagej.nih.gov/ij/

### CONTACT FOR REAGENT AND RESOURCES SHARING

Requests for reagents and resource sharing should be directed to the Lead Contact, Bo Zhao (zhaozb@iupui.edu).

### EXPERIMENTAL MODEL AND SUBJECT DETAILS

#### Cell culture

HEK293 and COS7 cells were obtained from ATCC. Cells were maintained in the DMEM medium (Invitrogen) supplemented with 10% heat-inactivated fetal bovine serum, 1% penicillin/streptomycin. All cells were grown at 37°C in a 5% CO_2_ humidified atmosphere.

#### Animal Models and Animal Care

*Grxcr2*^D46/D46^, *Grxcr2^D85/D85^* and *taperin^In103/In103^* mice were made on a C57BL/6J background using CRISPR/Cas9 technology. The exon 1 sequence of *Grxcr2* and *taperin* were analyzed by using the CRISPR design tool (https://crispr.med.harvard.edu/sgRNAScorer/). To generate *Grxcr2^D46/D46^* and *Grxcr2^D85/D85^* mice, the sgRNA-target genomic DNA sequence 5′- GGATGGCGTTTATGGGTCTGGGG –3′ and 5′- CAGCGGCGCCTACACTCTGGCGG –3′ were chosen. To generate *taperin^In103/In103^* mice, the sgRNA-target genomic DNA sequence 5′- GGATCTGGAGCGGCGCCGGATGG –3′ and 5′- CGACGGCACTGCCGGCCCCGGGG –3′ were chosen. Chimeric guide RNAs were synthesized by *in vitro* transcription following the published protocol (Yang et al., 2014). One-cell embryos were then microinjected by the genomics facility at The Scripps Research Institute. Genomic DNA was then collected from the offspring obtained by the embryo injections and screened using PCR and sequencing to determine mutations. The founder mice were then backcrossed with C57BL/6J mice for two generations. For genotyping of the *Grxcr2^D46/D46^* and *Grxcr2^D85/D85^* mice, the following primers were used: 5′- TCTTCCTACAGTGGCCGAGT –3′ and 5′- TGAATGTGAGCGAGATACCG –3′. For genotyping *taperin^In103/In103^* mice, the following primers were used: 5′-CTGGAAACGGGAGATCCTTG –3′ and 5′- GAAGCCTGGCGCTGACTC –3′. Both male and female mice were used in our experiment, and we did not find any sex-based differences.

All animal experiments were approved by Institutional Animal Care and Use Committee of The Scripps Research Institute and Indiana University School of Medicine.

### METHOD DETAILS

#### ABR measurement

To evaluate the extent to which the mutations induced hearing loss in these mice, ABR experiments were performed, as described previously, using TDT Bioacoustic system 3 and software (BioSig) (Zhao et al., 2014, 2016). In brief, mice were anesthetized using the mixture of xylazine and ketamine. Electrodes were inserted under the skin at the vertex and ipsilateral ear, while a ground was inserted under the skin near the tail. The speaker was placed 5 cm away from the mouse ear. The intensity of sound stimulus was started at 90 dB and decreased stepwise to a sub-threshold level. ABR thresholds were analyzed for both ears and for a range of frequencies (for Pure Tone, 4–28 kHz). If no ABR wave was detected at maximum intensity stimulation, a nominal threshold of 90 dB was assigned. Both male and female mice were used in our experiment, and we did not find any sex-based differences.

#### Immunostaining

Cochlear whole mount staining and immunocytochemistry of COS7 cells were carried out as described (Zhao et al., 2014, 2016). In brief, cochlear shells were dissected, opened and incubated in the fixative containing 4% PFA in Hank’s Balanced Salt Solution (HBSS) for 20 min. After washed in HBSS, the cochlear shell, Reissner’s membrane and the tectorial membrane were removed. Tissues were blocked for 20 min at room temperature in HBSS containing 5% bovine serum albumin (BSA), 1% goat serum and 0.5% Triton X-100, and then incubated overnight at 4°C with primary antibodies in HBSS containing 1% BSA and 0.1% Triton X-100. Tissues were washed in HBSS and incubated 2 hours at room temperature with secondary antibodies. Tissues were mounted in ProLong^®^ Antifade Reagents (Invitrogen). Stacked images were then captured by fluorescence deconvolution microscope (Leica). The length of the tallest row of stereocilia of IHCs was measured by measuring the distance from the distal end to the taper region of stereocilia, which were revealed by phalloidin and taperin or GRXCR2 staining. To reduce the angle of stereocilia induced variation, stacked images were captured with 0.15 mm step size. Then, stereocilia with their base and distal end at the same image plane were chosen and their length was measured using software (ImageJ).

Primary antibodies were as follows: anti-GRXCR2 (rabbit, Sigma); anti-taperin (rabbit, Sigma); anti-HA (mouse, Cell signaling); anti-Myc (rabbit, Cell signaling). Additional reagents were: Alexa Fluor 488-phalloidin (Invitrogen), Alexa Fluor 568-phalloidin (Invitrogen), Alexa Fluor 647-phalloidin (Invitrogen), Alexa Fluor 488 goat anti-rabbit (Invitrogen), Alex Fluor 546 goat anti-rabbit (Invitrogen) and Alexa Fluor 555 goat anti-mouse (Invitrogen).

#### Scanning electron microscopy

The experiment was performed as described (Zhao et al., 2014, 2016). In brief, inner ears were dissected in fixative (2.5% glutaraldehyde; 4% formaldehyde; 0.05 mM HEPES Buffer pH 7.2; 10 mM CaCl_2_; 5 mM MgCl_2_; 0.9% NaCl) and fixed for 1 hour at RT. Samples were then dissected to remove the stria vascularis, Reissner’s membrane and tectorial membrane. Samples were post-fixed by immersion in for 1 day in the same fixative at 4°C and washed by washing buffer (0.05 mM HEPES Buffer pH 7.2; 0.9% NaCl). After fixed in 1% OsO_4_for 1 hour, samples were serially dehydrated in ethanol, dried in a critical point drier (Autosamdri-815A, Tousimis), fine dissected and mounted on aluminum stubs. Samples were then coated by gold and viewed on a JEOL 7800F scanning electron microscope. At least three individual animals representative of each experimental paradigm were analyzed.

#### DNA constructions, immunoprecipitations and western blots

The coding sequence of *Grxcr2* and *taperin* were amplified from mouse cochlear cDNA library and cloned into the pEGFP-N3-derived vector, in which the EGFP coding region was deleted, or substituted by HA-tag or Myc-tag coding sequence. Expression of the constructs, immunoprecipitations, and western blots were carried out as described (Zhao et al., 2014, 2016). Immunoprecipitation experiments were carried out at least 3 times to verify the reproducibility of the data. The following antibodies were used for the experiments: anti-HA (mouse, Cell signaling); anti-Myc (rabbit, Cell signaling); anti-Myc (mouse, Santa Cruz); anti-GFP (mouse, Santa Cruz).

#### Injectoporation

The experiment was performed as described (Xiong et al., 2014; Zhao et al., 2014, 2016). In brief, the organ of Corti was isolated and placed in DMEM/F12 medium with 1.5 μg/ml ampicillin. For electroporation, glass electrodes (2 μmm diameter) were used to deliver the plasmid (500 ng/μl in 1× HBSS) to the sensory epithelium. A series of 3 pulses were applied at 1 s intervals with a magnitude of 60V and duration of 15 msec (ECM 830 square wave electroporator; BTX). Two days after injectoporation, samples were fixed and immunostaining was performed.

### QUANTIFICATION AND STATISTICAL ANALYSIS

#### Data analysis

All data are mean ± SEM. Student’s two-tailed unpaired t test or Two-way ANOVA were used to determine statistical significance (*, p < 0.05, **, p < 0.01, ***, p < 0.001).

## Supplementary Material

1

## Highlights

- GRXCR2 and taperin are essential for the morphogenesis of stereocilia and hearing
- GRXCR2 is essential for the localization of taperin to the basal region of stereocilia
- Reducing taperin expression restores morphological defects of *Grxcr2*-deficient hair cells
- Reducing taperin expression restores hearing loss caused by loss of GRXCR2
