# Supplementary material for: GRXCR2 Regulates Taperin Localization Critical for Stereocilia Morphology and Hearing
Source: Cell Rep. Author manuscript; Available in PMC 2019 Jan 3. (PMC6317715; doi:10.1016/j.celrep.2018.09.063)
Supplement: 1 [file NIHMS1511990-supplement-1.pdf]

Cell Reports, Volume *25*

Supplemental Information

GRXCR2 Regulates Taperin Localization Critical for Stereocilia Morphology and Hearing

Chang Liu, Na Luo, Chun-Yu Tung, Benjamin J. Perrin, and Bo Zhao

Figure S1

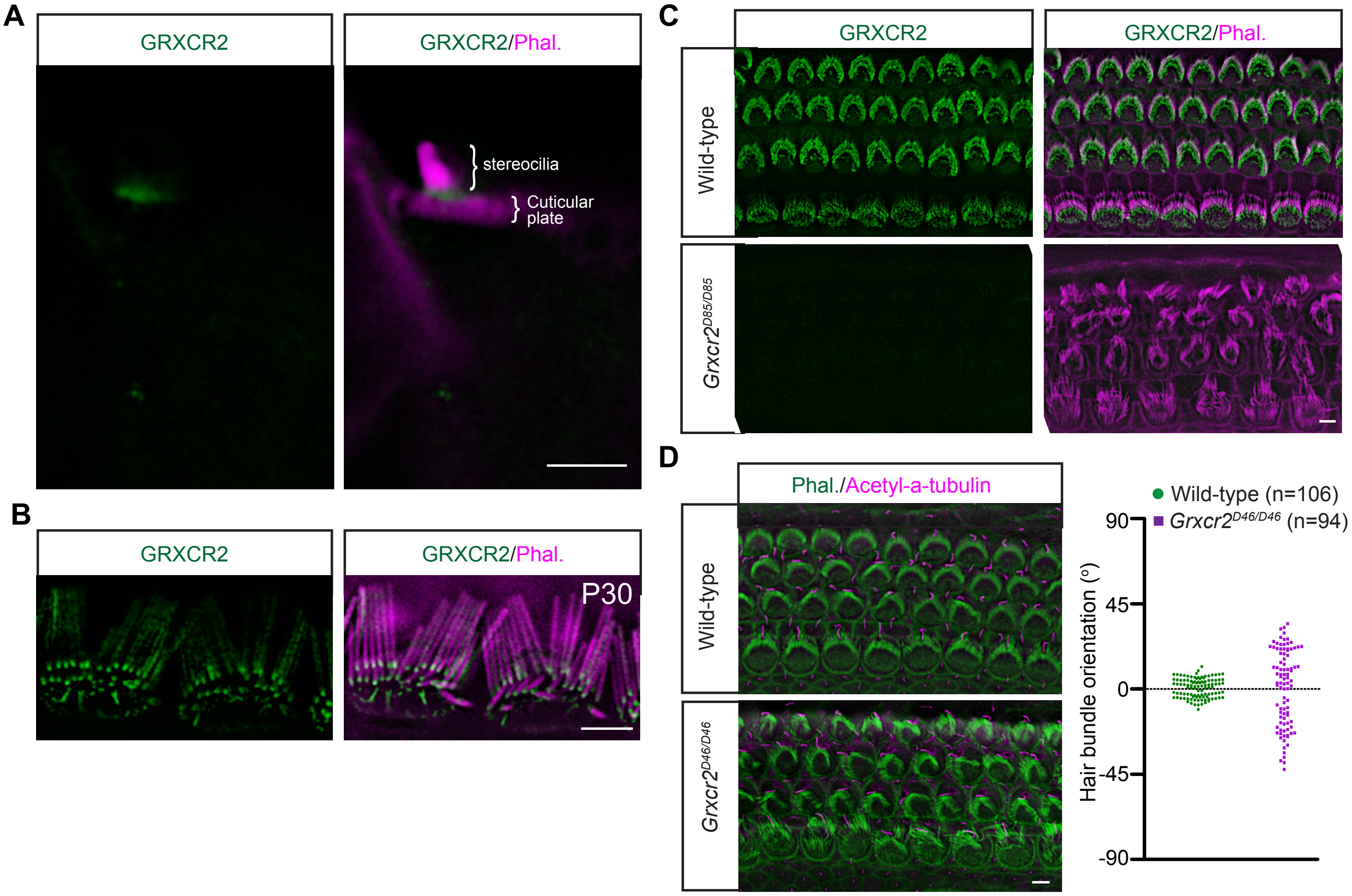
A
GRXCR2
GRXCR2/Phal.
stereocilia
Cuticular plate
B
GRXCR2
GRXCR2/Phal.
P30
C
GRXCR2
GRXCR2/Phal.
Wild-type
Grxcr2D85/D85
D
Phal./Acetyl-a-tubulin
Wild-type
Grxcr2D46/D46
Wild-type (n=106)
Grxcr2D46/D46 (n=94)
Hair bundle orientation (°)
90
45
0
-45
-90

Figure S1: GRXCR2 staining, related to Figure 1. (A) The inner ear section (P9) was stained with GRXCR2 antibody and phalloidin. Note, expression of GRXCR2 at the base of the stereocilia. **(B)** Cochlear whole mounts from wild-type mice at P30 were stained for GRXCR2 and phalloidin to reveal stereocilia. **(C)** Cochlear whole mounts from wild-type and *Grxcr2*$^{D85/D85}$ mice at P5 were stained for GRXCR2 and phalloidin to reveal stereocilia. Note the absence of a signal in the mutant mice with disorganized stereocilia. (D) Cochlear whole mounts at P2 were stained with phalloidin to reveal stereocilia and with an antibody to acetylated-α-tubulin to reveal kinocilia. Bundle orientation of the outer hair cells was measured as described (Zhao et al., 2016). Each dot represents one outer hair cell (n=106 for controls; n=94 for mutants). Scale bars: 5 µm.

Figure S2

A

B

C

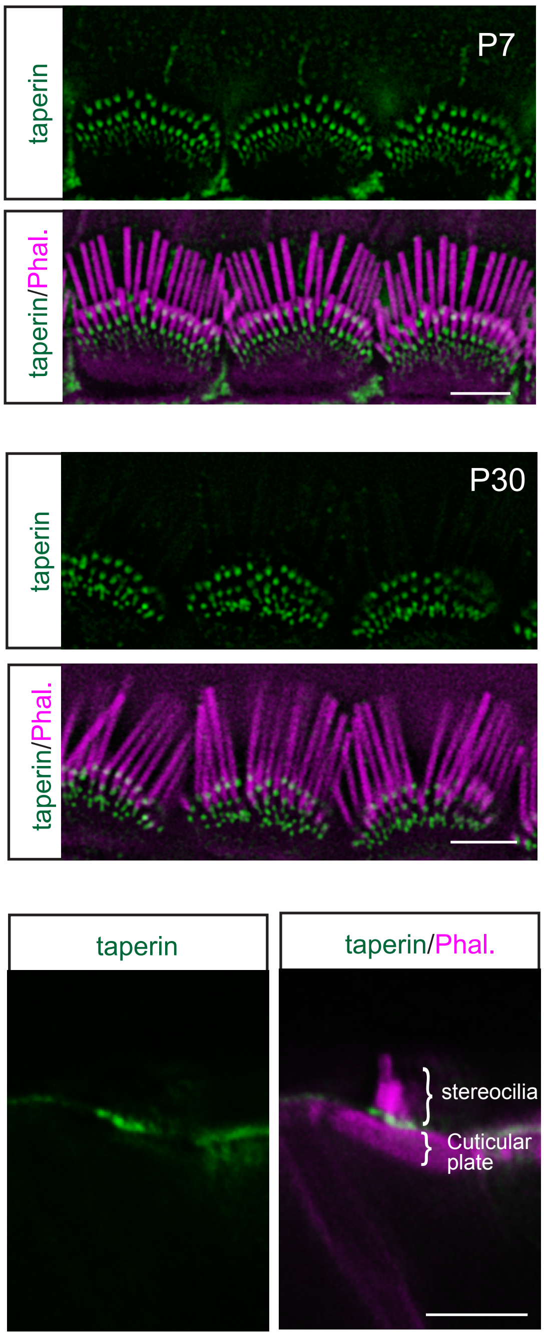

D

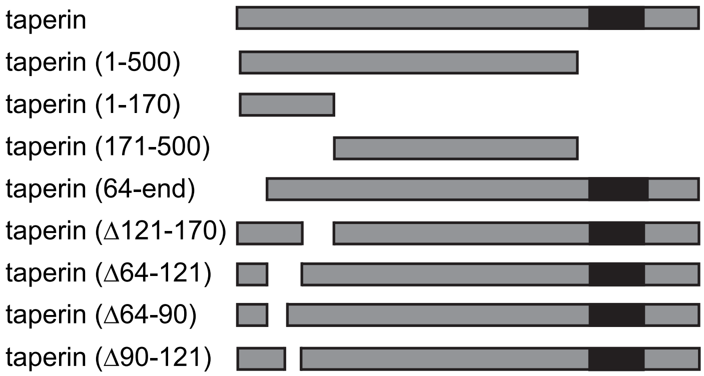

E

	Interaction with GRXCR2
taperin	+++
taperin (1-500)	+++
taperin (1-170)	+++
taperin (171-500)	–
taperin (64-end)	+++
taperin (Δ121-170)	+++
taperin (Δ64-121)	–
taperin (Δ64-90)	+++
taperin (Δ90-121)	+

F

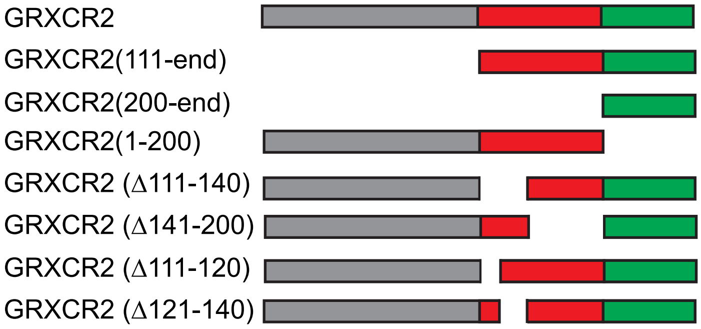

G

	Interaction with taperin
GRXCR2	+++
GRXCR2(111-end)	+++
GRXCR2(200-end)	–
GRXCR2(1-200)	+++
GRXCR2 (Δ111-140)	–
GRXCR2 (Δ141-200)	+++
GRXCR2 (Δ111-120)	+++
GRXCR2 (Δ121-140)	–

H

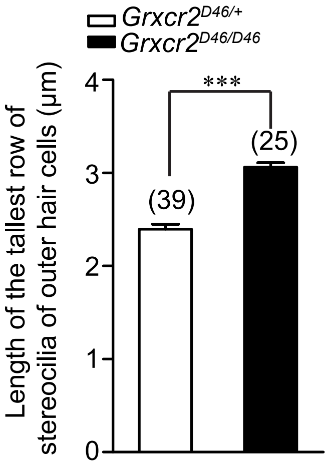

Figure S2: Related to Figure 2. (A-B) Costaining of P7 (A) and P30 (B) cochlear whole mounts with taperin-antibody (green) and phalloidin (magenta) to reveal stereocilia. Note the localization of taperin at the base of the stereocilia. **(C)** Inner ear section (P9) was stained with taperin antibody and phalloidin. Note, expression of taperin at the base of stereocilia. (D-G) Summaries of the Co-IP results. Diagram of taperin constructs (D) and GRXCR2 constructs (F) used for biochemical experiments. The region of taperin with higher similarity to phostensin is indicated in black (D). The glutaredoxin-like domain of GRXCR2 is indicated in red and cysteine-rich domain is indicated in green (F). A series of taperin (E) and GRXCR2 (G) mutants were co-immunoprecipitated with full-length GRXCR2 (E) or taperin (G). Their interactions were then analyzed by western blotting. Note, the 30-amino acids from 90-121 in taperin and 20-amino acids from 121-140 in GRXCR2 are important for their interactions. (H) Length of the tallest row of stereocilia of the outer hair cells. Cochlear whole mounts from P7 control and *Grxcr2*$^{D46/D46}$ animals were stained for taperin and phalloidin. The length of the tallest row of stereocilia was measured by ImageJ by measuring the distance from the distal end to the basal region of stereocilia, which was revealed by phalloidin and taperin staining. The number of analyzed inner hair cells is indicated in brackets. All values are the mean ± standard error of the mean (SE). Scale bars: 5 µm.

Figure S3

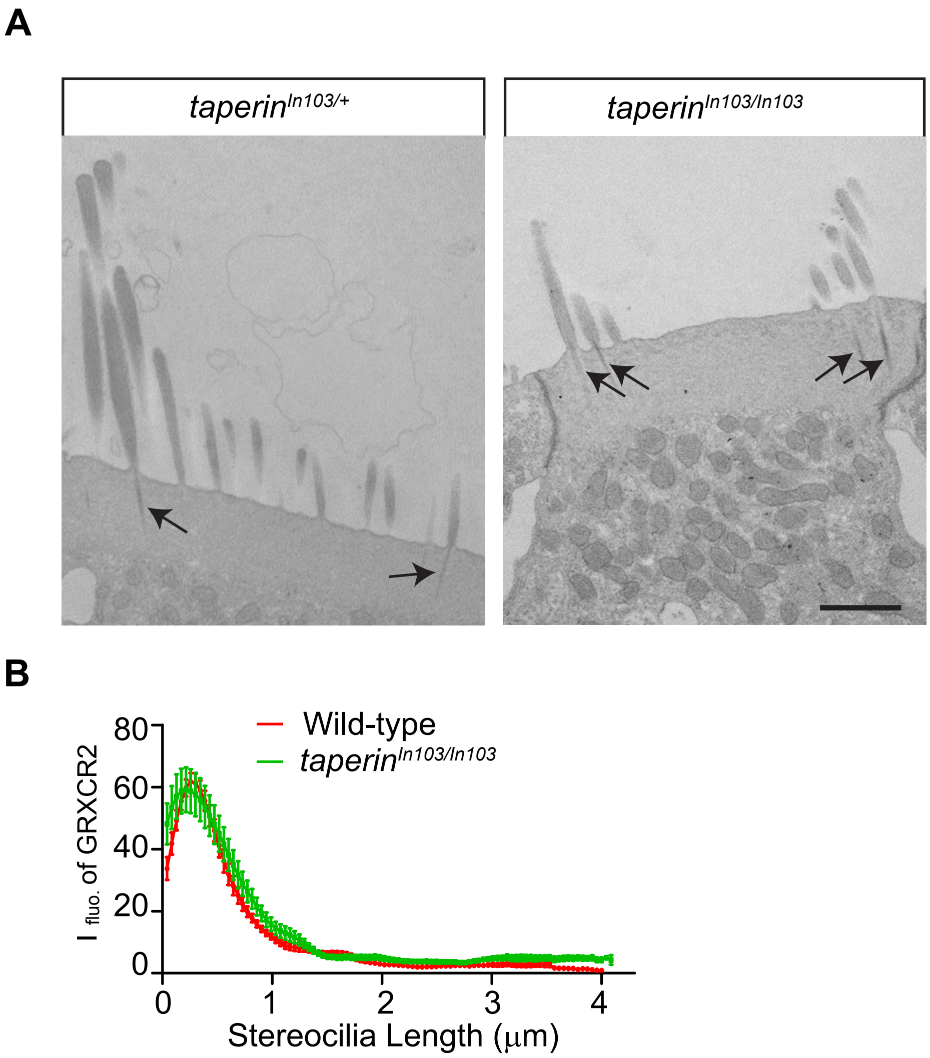

A
taperin^ln103/+
taperin^ln103/ln103
B
Wild-type
taperin^ln103/ln103
I fluo. of GRXCR2
80
60
40
20
0
0
1
2
3
4
Stereocilia Length (µm)

Figure S3: Rootlet in *taperin*$^{In103/In103}$ hair cells, related to Figure 3. (A) Hair cells in P7 control and *taperin*$^{In103/In103}$ animals were analyzed by TEM. Note, the rootlet was intact in the mutant hair cells. Scale bar: 1 µm. **(B)** Illustrated by GRXCR2 and phalloidin staining, single stereocilium from the tallest row of P7 hair cells (n=10) was selected, and the intensity of GRXCR2 staining from the basal region (left) to the distal end (right) of stereocilia was measured by ImageJ (NIH). GRXCR2 localization has no significant change in *taperin*$^{In103/In103}$ hair cells. All values are the mean ± SE.

Figure S4

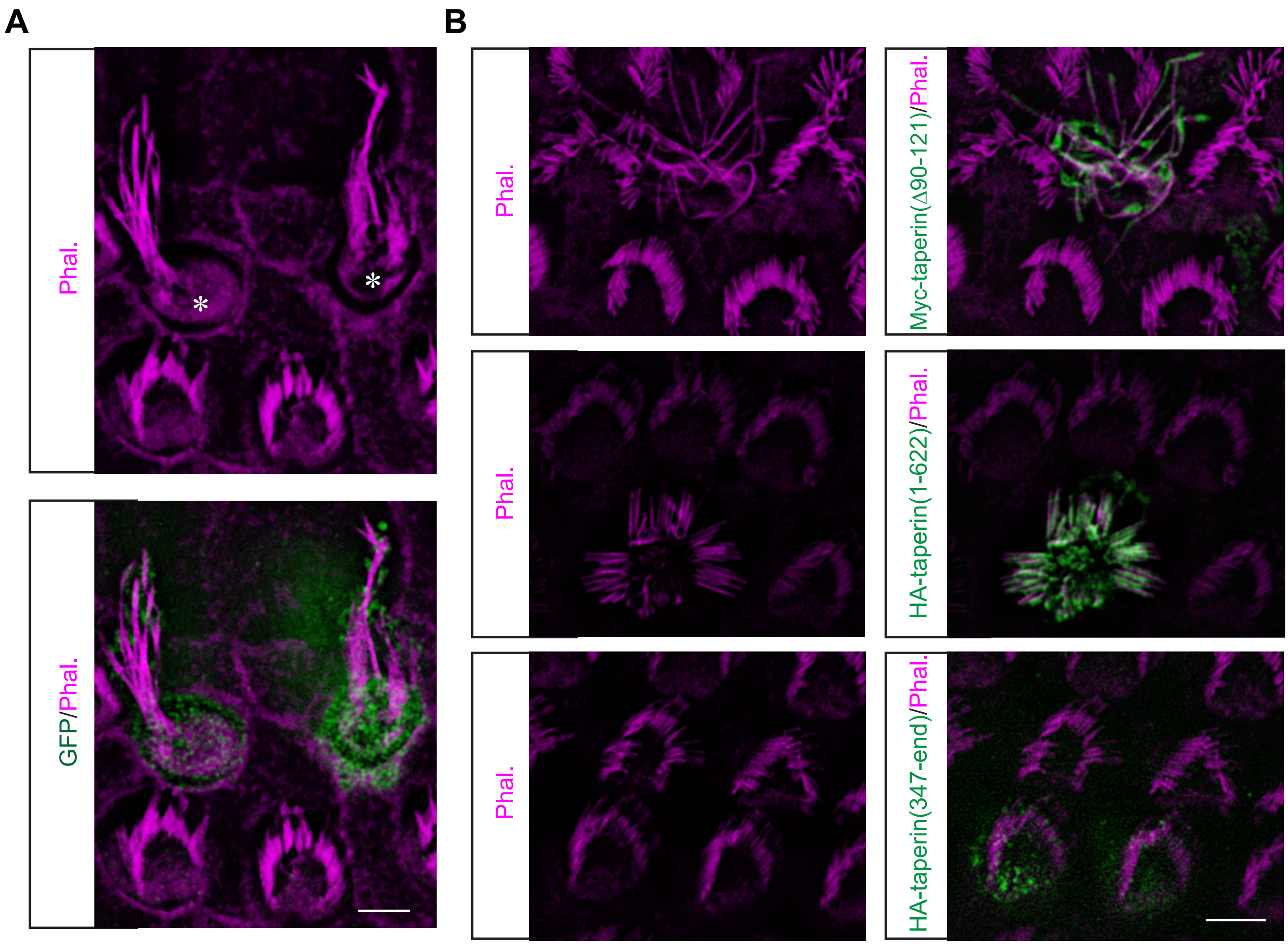

A
B
Phal.
GFP/Phal.
Myc-taperin(Δ90-121)/Phal.
HA-taperin(1-622)/Phal.
HA-taperin(347-end)/Phal.

Figure S4: Related to Figure 4. (A) Cochlear explants were prepared at P4 and injectoporated to coexpress untagged taperin and GFP. Two days later, tissues were fixed and stereocilia were visualized by phalloidin staining. Note, longer and disorganized stereocilia in the taperin injectoporated hair cells, indicated by GFP fluorescence (asterisks). (B) Injectoporation of Myc-taperin(Δ90-121), HA-taperin(1-622) and HA-taperin(347-end). Note, injectoporation of Myc-taperin(Δ90-121) and HA-taperin(1-622) led to the extraordinary growth of stereocilia. Scale bars: 5 µm.
